# Concurrent and prospective associations of obsessive-compulsive symptoms with suicidality in young adults: A genetically-informative study

**DOI:** 10.1016/j.jad.2020.10.065

**Published:** 2021-02-15

**Authors:** Georgina Krebs, David Mataix-Cols, Frühling Rijsdijk, Christian Rück, Paul Lichtenstein, Sebastian Lundström, Henrik Larsson, Thalia C. Eley, Lorena Fernández de la Cruz

**Affiliations:** a King's College London, MRC Social, Genetic and Developmental Psychiatry Centre, Institute of Psychiatry, Psychology & Neuroscience, London, United Kingdom; b National and Specialist OCD and Related Disorders Clinic for Young People, South London and Maudsley NHS Foundation Trust, London, UK; c Centre for Psychiatry Research, Department of Clinical Neuroscience, Karolinska Institutet, & Stockholm Health Care Services, Stockholm County Council, Stockholm, Sweden; d Department of Medical Epidemiology and Biostatistics, Karolinska Institutet, Stockholm, Sweden; e Gillberg Neuropsychiatry Centre, Centre for Ethics, Law and Mental Health, University of Gothenburg, Sweden; f School of Medical Sciences, Örebro University, Örebro, Sweden

**Keywords:** Obsessive-compulsive disorder, Suicidal ideation, Suicide attempts, Twin design, Genetic, Adolescence

## Abstract

**Background:**

Obsessive-compulsive disorder (OCD) has been linked with elevated risk of suicidality. However, most previous studies have been cross-sectional, and little is known about the aetiology of the association between obsessive-compulsive symptoms (OCS) and suicidality in young adults.

**Methods:**

Participants were members of the Child and Adolescent Twin Study in Sweden, at ages 18 (*n* = 9,162) and 24 (*n* = 3,466). Twins completed self-report measures, including assessment of OCS, suicidal ideation, and suicidal attempts. Logistic regression models tested concurrent and prospective associations of total OCS and OCS dimensions with suicidality, with and without adjustment for depression and anxiety symptoms. Genetic models tested the extent to which the main phenotypic associations were accounted for by genetic and environmental influences.

**Results:**

Total OCS were significantly associated with concurrent reports of suicidality at age 18 and 24, even when controlling for depressive and anxiety symptoms. Taboo obsessions (e.g., sexual and aggressive thoughts) were more robustly associated with suicidality than other OCS dimensions, and prospectively predicted suicidality symptoms over time, even when controlling for baseline suicide attempts. Genetic factors accounted for most of the concurrent and longitudinal covariance between OCS and suicidality, with substantial non-shared environmental influences.

**Limitations:**

We relied on self-report measures and did not include diagnostic assessment of OCD.

**Conclusions:**

OCS, particularly taboo obsessions, are associated with significantly elevated risk of suicidality in late adolescence and early adulthood. This relationship is explained by a combination of common genetic liability and non-shared environmental effects, suggesting that effective OCS treatment might reduce suicidality risk in this group.

## Highlights

• Obsessive-compulsive symptoms (OCS) are associated with risk of suicidality in youth.
• Taboo obsessions are more robustly linked with suicidality than other OCS.
• OCS in late adolescence prospectively predict suicidality in early adulthood.
• Genetic factors explain much of the relationship between OCS and suicidality.
• Non-shared environmental factors also contribute to the relationship.

## 1 Introduction

Suicide is a major public health concern and the second leading cause of death amongst adolescents and young adults globally (Centers for Disease Control and Prevention, 2017; Patton et al., 2009; World Health Organization, 2014). The most robust predictors of death by suicide include suicidal ideation, self-harm, and suicide attempts (Hawton and Harriss, 2007). Identifying factors underpinning suicidality in the broader sense is therefore crucial to informing suicide prevention strategies.

Increasing evidence has indicated that obsessive-compulsive disorder (OCD) is associated with elevated risk for suicidality (Angelakis et al., 2015; Fernández de la Cruz et al., 2017; Pellegrini et al., in press; Sidorchuk et al., 2019). In the two largest studies to date, data from Swedish national registers showed OCD to be associated with approximately a five-fold increased risk of attempting suicide (Fernández de la Cruz et al., 2017; Sidorchuk et al., 2019). This relationship was not fully accounted for by coexisting psychopathology, suggesting that OCD is an independent risk factor for suicidality. Despite the rigour of these studies, findings pertain to individuals with OCD diagnosed in specialist care settings and documented suicide attempts leading to clinical care. Since healthcare utilisation and clinical detection is low for both OCD (Kohn et al., 2004) and suicidality (De Leo et al., 2005), there is a need for further studies using systematic screening in community populations. Furthermore, obsessive-compulsive symptoms (OCS) are known to occur on a continuum (Olatunji et al., 2008), with up to 17% of the population experiencing sub-threshold symptoms that are impairing and associated with significant psychiatric comorbidity (de Bruijn et al., 2010; Fullana et al., 2009; Grabe et al., 2000). Investigating the association with suicidality across the OCS continuum is thus of both theoretical and clinical importance.

Additionally, there are several notable gaps in the literature on OCD and suicidality. First, the direction of the association has rarely been tested (Pellegrini et al., in press), despite the impact such knowledge could have on suicidality prevention strategies. There is preliminary evidence that OCS severity prospectively predicts suicidal ideation amongst adults with OCD (Brown et al., 2019), but further investigation is required.

Second, most studies have focused on *adult* samples (Pellegrini et al., in press). Little attention has been given to the relationship between OCD and suicidality amongst *young people*. Such research is crucial given that suicidal thoughts and behaviours often emerge during youth (Piscopo et al., 2016), whereas death by suicide peaks in mid-adulthood (McKeown et al., 2006). Hence, there may be greatest potential for suicide prevention strategies during adolescence and early adulthood.

Third, the association of different OCD symptom dimensions with suicidality remains unclear. OCD encompasses a number of relatively independent symptom dimensions (Bloch et al., 2008; Mataix-Cols et al., 2005, 2008), which have distinct aetiologies, neural substrates, and clinical correlates (Iervolino et al., 2011; Mataix-Cols et al., 2005, 2008). There is some suggestion that suicidality may be especially linked to taboo obsessions, such as sexual, aggressive, and religious obsessions (Balci and Sevincok, 2010; Khosravani et al., 2017; Kim et al., 2016; Storch et al., 2015; Torres et al., 2011; Velloso et al., 2016), but findings have been mixed and further clarification is needed (Pellegrini et al., in press).

Finally, little attention has been given to the mechanisms underpinning the relationship between OCD and suicidality. Both are under genetic influence (Maciejewski et al., 2014; Pauls, 2008) so their association could be due to shared genetic vulnerability. Alternatively, the relationship could reflect common environmental risk and/or suicidality could arise as a functional consequence of the psychosocial burden of experiencing OCD. To the best of our knowledge, only one previous study has addressed this question (Sidorchuk et al., 2019). Using genetic modelling of family data, this study found that the association between OCD and suicide attempts was explained by additive genetic factors (60%) and environmental experiences that are specific to individuals (i.e., non-shared environment; 40%). Of note, this study included a wide participant age range (10–44 years at the end of the follow-up period). Further research is needed to establish whether there are developmental differences in the aetiology of the covariance between OCS and suicidality, especially given that genetic influences on OCS have been shown to be developmentally dynamic (Krebs et al., 2014). Moreover, the aetiology of the *prospective* association between OCS and suicidality remains unknown, despite its clinical importance.

In summary, the extent and aetiology of the associations between OCS and suicidality remain unclear. The current study utilised data from a large, population-based twin sample of adolescents and young adults and addressed four related questions. First, we sought to establish the cross-sectional associations between total OCS and suicidality. Second, we explored differential links of specific OCS dimensions with suicidality. Third, we tested the extent to which OCS prospectively predict changes in suicidality. Fourth, we estimated the extent to which cross-sectional and longitudinal associations between OCS and suicidality are explained by genetic and environmental factors.

## 2 Method

### 2.1 Study population

Data were drawn from the Child and Adolescent Twin Study in Sweden (CATSS; Anckarsäter et al., 2011), an ongoing longitudinal study of all twins born in Sweden since July 1992. Our analyses focused on data obtained when the twins were 18 (CATSS-18) and 24 years old (CATSS-24). At both ages, twins completed OCS questionnaires and items relating to suicidality. At age 18, parents/caregivers also reported on suicidality in their child. At the time of the analyses, data on OCS and suicidality were available for 9162 CATSS-18 participants and 3466 CATSS-24 participants. Demographic and clinical characteristics are shown in Table 1. A sub-sample of participants (*n* = 2226) had OCS and suicidality data at *both* ages. Compared to those with data at age 18 only, these participants were more likely to be female (57.2% vs 52.9%, respectively), and reported greater depressive symptoms (8.67 vs 8.43, respectively) and lower anxiety symptoms (2.50 vs 2.58, respectively) at age 18 (see Table S1). Of note, OCS and suicidality at age 18 were not associated with whether measures were completed at both ages (see Table S1).

**Table 1:** Demographic and clinical characteristics of the sample at ages 18 and 24.

	Cohort	
	CATSS-18	CATSS-24
	(*N* = 9162)	(*N* = 3466)
Sex, *n* (%)		
Male	3775 (41.2%)	1482 (42.8%)
Female	5387 (58.8%)	1984 (57.2%)
Zygosity, *n* (%)		
Monozygotic	2698 (29.5%)	1106 (31.9%)
Dizygotic	6101 (66.6%)	2256 (65.1%)
Unknown	363 (4.0%)	104 (3.0%)
BOCS total, mean (SD)	1.82 (2.24)	–
CES-D total, mean (SD)	8.46 (6.02)	–
SCARED total, mean (SD)	15.93 (11.45)	–
OCI-R total, mean (SD)		8.01 (6.99)
HADS-depression, mean (SD)	–	5.26 (3.57)
HADS-anxiety, mean (SD)	–	6.64 (3.82)
Suicidality, *n* (%) endorsed		
Self-reported suicidal ideation	–	858 (24.2%)
Self-reported suicide attempts	593 (6.0%)	187 (5.29%)

*Note:* CATSS = Child and Adolescent Twin Study in Sweden; BOCS = Brief Obsessive Compulsive Scale; OCI-*R* = Obsessive-Compulsive Inventory-Revised version; CES-*D* = centre for Epidemiologic Studies Depression Scale; SCARED = Screen for Child Anxiety Related Emotional Disorders; HADS = Hospital Anxiety and Depression Scale. Summary statistics are presented on untransformed variables for comparison with other published samples.

Twin zygosity was determined by a panel of 48 single-nucleotide polymorphisms (SNPs) derived for zygosity analyses (Hannelius et al., 2007). If DNA was unavailable, an algorithm based on five questions of twin similarity was used, as previously described (Anckarsäter et al., 2011). Ethical approval for CATSS was granted by the Stockholm Regional Ethics Review Board (CATSS-18: reference number 2010/1410/31/1; CATSS-24: reference number 2015/1947–31/4).

### 2.2 Measures

#### 2.2.1 Obsessive-compulsive symptoms

At age 18, adolescents completed the 15-item symptom checklist of the Brief Obsessive Compulsive Scale (BOCS; Bejerot et al., 2014). The BOCS is based on the clinician-administered Yale-Brown Obsessive-Compulsive Scale (Goodman et al., 1989). Participants rated their experience of each symptom as ‘never’, ‘past’ or ‘current’; the latter two were combined for our analyses. The BOCS symptom checklist has high internal consistency and good sensitivity/specificity in discriminating OCD from other psychiatric disorders (Bejerot et al., 2014). A five-factor structure has been identified (Bejerot et al., 2014). In the current study, three items were excluded prior to analysis (hoarding, dysmorphic concerns, and self-injurious behaviour) because they assess related phenomena rather than the core OCD phenotype (Bejerot et al., 2014). These exclusions resulted in incomplete subscales, so a principal component analysis was conducted from tetrachoric correlations of the 12 remaining items, yielding a four-factor solution (see Table S2). These factors corresponded closely to the forbidden thoughts, symmetry, contamination, and magical thoughts subscales previously identified (Bejerot et al., 2014). Internal consistency was good for the BOCS total score (Cronbach's α=0.77), although lower for subscales (Cronbach's α of 0.70 for contamination; 0.53 for forbidden thoughts; 0.65 for symmetry; and 0.42 for magical thoughts).

At age 24, participants completed the Obsessive-Compulsive Inventory-Revised version (OCI-R). The OCI-R is an 18-item measure of distress associated with OCD symptoms in the previous month, with items rated on a five-point Likert scale (0=not at all, 4=very much). It has good internal consistency, convergent validity, and test–retest reliability in patients with OCD, anxiety disorders, and healthy controls (Foa et al., 2002). The measure comprises six, three-item subscales: washing, checking, ordering, obsessing, hoarding, and neutralizing. In CATSS-24, the hoarding and neutralizing subscales were not administered. Therefore, participants completed 12 OCI-R items, yielding a total score ranging from 0 to 48. Internal consistency was good for the OCI-R (Cronbach's α=0.86) and its subscales (Cronbach's α of 0.76 for washing; 0.73 for checking; 0.79 for ordering; and 0.84 for obsessing).

#### 2.2.2 Suicidality

Suicidality was assessed with a range of items (three items at age 18 and five at age 24; see Table S3 for full details). Consistent with a previous study using these data (Krebs et al., in press), items selected for the main analyses at both ages were chosen because they corresponded closely to established definitions of suicidal ideation and attempts (Silverman et al., 2007). At age 18, a self-reported suicide attempt item was selected, while at age 24, a suicidality composite was created in order to maximise statistical power. The composite combined a self-reported suicidal ideation item and suicide attempt item. The composite was coded as a binary outcome (yes/no), meaning that an individual scored positive if they endorsed either of the two constituent items. A suicidality composite was not created at age 18 because only one self-report item was available. Self- and parent-reported items were not combined since: a) parents typically underreport suicidality (Breton et al., 2002); and b) parent-reported items did not correspond as well to recognised definitions of suicidal ideation and attempts (Silverman et al., 2007). Phenotypic analyses were repeated with all individual suicidality items.

#### 2.2.3 Depressive and anxiety symptoms

At age 18, depressive symptoms were assessed using the Iowa version of the centre for Epidemiologic Studies Depression Scale (CES-D; Kohout et al., 1993). This self-report measure comprises 11 items scored on a 4-point scale ranging from 0 (rarely/none of the time) to 3 (most/all of the time), giving a total score between 0 and 33. The scale has good psychometric properties and correlates highly with the original CES-D (Carpenter et al., 1998). In the current study, internal consistency for the CES-D was good (Cronbach's α=0.87).

Anxiety was assessed at age 18 using the 38-item, self-report version of the Screen for Child Anxiety Related Emotional Disorders (SCARED; Birmaher et al., 1997). This measure includes items reflecting the five common anxiety diagnoses, with items scored on a 3-point scale ranging from 0 (almost never true) to 2 (true most of the time), yielding a total score ranging from 0 to 76. The SCARED has good psychometric properties (Birmaher et al., 1997; Hale et al., 2005; Monga et al., 2000). Internal consistency in the current sample was excellent (Cronbach's α=0.93).

At age 24, the CES-D and the SCARED were not administered, but instead anxiety and depressive symptoms were assessed using the Hospital Anxiety and Depression Scale (HADS; Zigmond and Snaith, 1983). The HADS is a 14-item questionnaire, comprising a 7-item general anxiety subscale (HADS-A) and a 7-item depression subscale (HADS-D). Items are scored on a 4-point scale ranging from 0 (never) to 3 (almost always). The HADS is psychometrically robust (Bjelland et al., 2002) and, in the current study, internal consistency was good (Cronbach's α=0.83 and 0.79 for the HADS-A and the HADS-D, respectively).

### 2.3 Statistical analysis

#### 2.3.1 Phenotypic analyses

The association between OCS severity and suicidality was examined using a series of logistic regression models. These tested the extent to which total OCS were associated with suicide attempts at age 18 and suicidality at age 24, with and without adjustment for co-occurring symptoms of depression and anxiety. Analyses were then repeated simultaneously including all OCS subscale scores, in order to investigate the link between different OCS dimensions and suicidality. Lastly, the prospective association between OCS (at age 18) and suicidality (at age 24) was examined, first with just those variables, then also controlling for suicide attempts reported at age 18. Finally, longitudinal analyses were repeated, including adjustment for symptoms of depression and anxiety at age 18.

Logistic regressions were conducted in STATA version 14.2, using the robust cluster option to account for the non-independence of twins/siblings. The BOCS, OCI-R, CES-D, and SCARED showed evidence of positive skew and were therefore log-transformed prior to analyses (see Table S4). In addition, continuous variables were standardised by rescaling variables to have a mean of 0 and standard deviation of 1, for ease of comparison across scales. All regression models controlled for age and sex.

#### 2.3.2 Genetic analyses

The aetiology of the associations between OCS and suicidality was explored using twin models. We planned *a priori* to conduct these analyses for phenotypic associations >0.2, given that decomposition of smaller associations typically leads to unstable solutions and/or imprecise parameter estimates. The twin design compares the degree of phenotypic similarity between monozygotic (MZ) twins, who share 100% of their genes, and dizygotic (DZ) twins, who share on average 50% of their segregating genes (Rijsdijk and Sham, 2002). Within-pair correlations for MZ twins are compared with those for DZ twins. Greater MZ compared to DZ phenotypic similarity is attributed to additive genetic effects (A). Within-pair similarity that is not accounted for by genetic factors is attributed to shared environmental effects (C). Non-shared environmental effects (E) are estimated from the within-pair differences between MZ twins, which also includes measurement error. The same principles can be extended to multivariate twin models, in order to estimate the aetiology of associations between variables. Multivariate models are based on cross-twin cross-trait correlations (e.g., the correlation between twin 1′s score on the first trait and twin 2′s score on the second trait). A higher cross-twin cross-trait correlation for MZ compared to DZ twins indicates that genetic factors have a degree of influence on the phenotypic variance shared by two traits.

The concurrent phenotypic association between OCS and suicidality at both ages was decomposed into genetic and environmental influences using the correlated factor solutions of a bivariate Cholesky decomposition model. The prospective association between OCS at age 18 and suicidality at age 24, accounting for suicidality at age 18, was examined with a trivariate Cholesky decomposition model. Within this model, we used a correlated factors solution to represent the concurrent association between suicidality and OCS at age 18, given there is no confirmed direction of effect between these variables. However, the *prospective* associations of suicidality and OCS at age 18 with suicidality at age 24 were interpreted as a Cholesky decomposition paths, allowing genetic and environmental effects on suicidality and OCS at age 18 to influence suicidality at age 24, but not vice versa. For all models, ordinal variables (suicidality and BOCS subscale scores) were modelled using liability thresholds. Liability threshold models assume that ordered categories (e.g., the presence or absence of suicidality) reflect an imprecise measurement of an underlying normal distribution of liability, with one or more thresholds discriminating between the different categories (Rijsdijk and Sham, 2002).

Genetic modelling was conducted within R using OpenMx (Boker et al., 2011). Continuous OCS scores were sex regressed prior to analysis to avoid artificial inflation of MZ versus DZ correlations (McGue and Bouchard, 1984). For ordinal variables, age and sex effects were included as covariates in the threshold equations. Models were fitted using raw data full information maximum likelihood. The fit statistic provided by OpenMx is minus twice the log likelihood (−2LL) of the observations, which provides a relative measure of fit. The difference in −2LL (which is Chi-square distributed) and difference in degree-of-freedom between models is used to examine the overall fit of a model. We also examined model fit using Akaike information criterion (AIC), with lower values indicating a better balance between explanatory power and parsimony. A difference in AIC ≥3 indicates support for the lower AIC model (Burnham and Anderson, 1998). Significance of parameters is established by 95% maximum likelihood confidence intervals (CI).

## 3 Results

### 3.1 Phenotypic findings

Logistic regression models showed that total OCS were positively associated with concurrent measures of suicide attempts at age 18 and suicidality at age 24 (see Table 2). Although these associations decreased when controlling for co-occurring depression and anxiety symptoms, they remained significant. Analysis of additional suicidality items showed a similar pattern of results, with total OCS being associated with all aspects of suicidality at ages 18 and 24 (see Table 3). These associations were more robust for self-reported suicidality items and the association of OCS with parent-reported suicidal ideation and attempts at age 18 did not remain significant when controlling for depressive symptoms.

**Table 2:** Results of logistic regression models testing cross-sectional associations between obsessive-compulsive symptoms and main suicidality outcome at age 18 and 24.

		Odds ratios		
		Without adjustment	With adjustment for depression	With adjustment for depression and anxiety
AGE 18				

**Model 1**	BOCS total	1.84 (1.68 – 2.01)⁎⁎⁎	1.39 (1.25 – 1.53)⁎⁎⁎	1.34 (1.20 −1.49)⁎⁎⁎
**Model 2**	Symmetry	1.30 (1.22 – 1.40)⁎⁎⁎	1.18 (1.09 – 1.27)⁎⁎⁎	1.17 (1.08 – 1.26)⁎⁎⁎
	Forbidden thoughts	1.55 (1.39 – 1.73)⁎⁎⁎	1.25 (1.11 – 1.40)⁎⁎⁎	1.21 (1.07 – 1.37)⁎⁎
	Contamination	1.09 (0.97 – 1.23)	1.06 (0.93 – 1.20)	1.04 (0.92 – 1.19)
	Magical thoughts	0.93 (0.77 – 1.12)	0.90 (0.74 – 1.10)	0.93 (0.76 – 1.14)

AGE 24				

**Model 1**	OCI-R total	1.81 (1.65 – 2.00)⁎⁎⁎	1.46 (1.32 – 1.61)⁎⁎⁎	1.22 (1.09 – 1.35)⁎⁎⁎
**Model 2**	Ordering	1.12 (1.00 – 1.25)*	1.07 (0.96 – 1.20)	1.04 (0.93 – 1.17)
	Obsessing	2.47 (2.24– 2.73)⁎⁎⁎	2.01 (1.82– 2.24)⁎⁎⁎	1.77 (1.58 – 1.97)⁎⁎⁎
	Washing	0.95 (0.87 – 1.05)	0.95 (0.86 – 1.04)	0.95 (0.86 – 1.05)
	Checking	0.82 (0.73 – 0.91)⁎⁎⁎	0.83 (0.74 – 0.93)⁎⁎	0.81 (0.72 – 0.91)⁎⁎⁎

Note: BOCS = Brief Obsessive Compulsive Scale; OCI-*R* = Obsessive-Compulsive Inventory-Revised version. BOCS subscale scores were ordinal and therefore not standardized. OCI-R subscale scores were standardized for ease of comparison. Sample size for analyses ranged from 8080 to 9162 at age 18, and 3408 to 3466 at age 24. All regression models controlled for age, sex and relatedness of twin members using robust clustering. 95% confidence intervals in parentheses.

⁎ *p* <. 05.

⁎⁎ *p* < .01.

⁎⁎⁎ *p* < .001.

**Table 3:** Results of logistic regression models testing the cross-sectional associations between total OCD symptoms and additional suicidality items at age 18 and 24.

		Odds ratios		
		Without adjustment for depression and anxiety	With adjustment for depression	With adjustment for depression and anxiety
AGE 18				

	Suicidal ideation (parent-report)	1.72 (1.41 – 2.11)⁎⁎⁎	1.17 (0.93 – 1.47)	1.13 (0.90 – 1.42)
	Suicide attempts (parent-report)	1.75 (1.39 – 2.21)⁎⁎⁎	1.13 (0.87 – 1.47)	1.06 (0.82 – 1.38)

AGE 24				

	Desire to be dead (self-report)	4.00 (2.92 – 5.48)⁎⁎⁎	2.33 (1.68 – 3.23)⁎⁎⁎	1.53 (1.08 – 2.17)⁎⁎⁎
	Suicidal ideation (self-report)	1.80 (1.63 – 1.98)⁎⁎⁎	1.44 (1.31 – 1.60)⁎⁎⁎	1.21 (1.09 – 1.35)⁎⁎⁎
	Suicide attempt (self-report)	2.10 (1.72 – 2.58)⁎⁎⁎	1.63 (1.34 – 1.98)⁎⁎⁎	1.27 (1.04 – 1.57)*
	Suicide attempt requiring medical attention (self-report)	2.47 (1.85 – 3.32)⁎⁎⁎	1.92 (1.43 – 2.57)⁎⁎⁎	1.51 (1.12 – 2.02)⁎⁎
	Suicide attempt requiring hospital admission (self-report)	2.31 (1.56 – 3.42)⁎⁎⁎	1.67 (1.14 – 2.47)⁎⁎	1.35 (0.91 – 2.01)

Note: Score on the Brief Obsessive Compulsive Scale and Obsessive-Compulsive Inventory-Revised were standardized for ease of comparison. Sample size for analyses ranged from 5538 to 9162 at age 18, and 3390 to 3466 at age 24. All regressions models controlled for age, sex and relatedness of twin members using robust clustering. 95% confidence intervals in parentheses.

⁎ *p* <. 05.

⁎⁎ *p* < .01.

⁎⁎⁎ *p* < .001.

Table 2 also includes the results of the logistic regression models testing the association of the OCS subscales with suicidality. At age 18, only the symmetry and forbidden thoughts subscales of the BOCS were uniquely associated with suicide attempts. These associations were attenuated but remained significant when adjusting for depression and anxiety symptoms. At age 24, the ordering, obsessing, and checking subscales of the OCI-R were uniquely associated with suicidality. Of note, while ordering and obsessing symptoms were positively associated with suicidality, checking symptoms showed a negative association. After adjustment for anxiety and depression, only obsessing and checking symptoms remained significantly associated with suicidality.

Table 4 shows the results of logistic regression models testing the prospective associations between OCS and suicidality. The first set of analyses focused on *total* OCS. Our results showed that total OCS at age 18 positively predicted suicidality at age 24, and this association remained significant even when controlling for suicide attempts at 18. However, this association became non-significant when adjusting for depression and anxiety at age 18. With regard to OCS subscales, symptoms of symmetry and forbidden thoughts at age 18 were positively associated with suicidality at age 24, even when adjusting for suicide attempts at age 18. However, when further adjusting for anxiety and depression at age 18, only forbidden thoughts predicted suicidality at age 24. Of note, in this analysis, symptoms of contamination emerged as a significant, negative predictor of suicidality at age 24.

**Table 4:** Results of logistic regression models testing prospective associations of obsessive-compulsive symptoms at age 18 with suicidality at age 24.

		Odds ratios			
		No adjustment for suicide attempts, depression, and anxiety at age 18	Adjustment for suicide attempts at age 18	Adjustment for suicide attempts and depression at age 18	Adjustment for suicide attempts, depression, and anxiety at age 18
**Model 1**	Total BOCS score	1.40 (1.26 - 1.55)⁎⁎⁎	1.31 (1.18 – 1.45)⁎⁎⁎	1.04 (0.92 – 1.19)	0.97 (0.85 −1.11)
**Model 2**	Symmetry	1.14 (1.03 – 1.26)*	1.12 (1.01 – 1.24)*	1.03 (0.92 – 1.15)	0.99 (0.87 – 1.12)
	Forbidden thoughts	1.54 (1.33 – 1.79)⁎⁎⁎	1.45 (1.24 – 1.68)⁎⁎⁎	1.24 (1.04 – 1.47)*	1.20 (1.01 - 1.43)*
	Contamination	0.98 (0.83 – 1.15)	0.96 (0.81 – 1.13)	0.81 (0.66 – 0.99)*	0.80 (0.65 - 0.99)*
	Magical thoughts	0.89 (0.67 – 1.16)	0.88 (0.66 −1.16)	0.97 (0.71 – 1.33)	0.91 (0.66 – 1.27)

Note: BOCS = Brief Obsessive Compulsive Scale. Sample size for analyses ranged from 1734 to 2226. All regression models controlled for age, sex and relatedness of twin members using robust clustering. 95% confidence intervals in parentheses.

⁎ *p* <. 05. ⁎⁎ *p* < .01.

⁎⁎⁎ *p* < .001.

### 3.2 Genetic findings

Bivariate twin analyses were run for OCS variables that had phenotypic correlations with suicidality >0.2 (i.e., total OCS at both ages; forbidden thoughts and symmetry at age 18; obsessional symptoms and ordering at age 24; see Table S5). For all analyses, full ACE models were initially estimated and compared to correlational models. For analyses in which estimates of C were non-significant and close to zero (i.e., below 5%), we tested more parsimonious AE models. In all of these instances, the AE model did not result in any significant loss of fit and was therefore selected as the final model. Model fits are shown in Tables S6 – S8.

Model estimates at ages 18 and 24 are shown in Table 5. For example, in the analysis of total OCS and suicide attempts at 18, heritability of each was 37% and 61%, with non-shared environmental factors accounting for the remaining 63% and 39% of the variance respectively. Of relevance to the current study, the genetic correlation between the total OCS and suicidality at 18 was moderate (rG = 0.42), indicating substantial overlap in the genetic factors underlying these phenotypes. In contrast, the non-shared environmental correlation was small (rG = 0.13), suggesting largely distinct environmental risk factors. Genetic influences accounted for 74.6% (95% CI 53.2–96.3) and non-shared environmental factors accounted for 25.4% (95% CI 3.7–46.8) of the association. A similar pattern of results was observed across other models, with moderate to large genetic correlations (ranging from 0.32 to 0.65) and small to moderate non-shared environmental correlations (ranging from 0.10 to 0.33). In all analyses, genetic factors accounted for a large and significant proportion of the phenotypic association between OCS and suicidality (estimates ranging from 48.2 to 74.6%). Non-shared environmental factors generally explained a significant, albeit smaller, proportion of the associations (estimates ranging from 21.0 to 50.4%). Shared environmental factors explained a small and non-significant proportion of the phenotypic association between symmetry symptoms and suicide attempts at age 18, and obsessing and suicidality at age 24.

**Table 5:** Estimates from bivariate models of obsessive-compulsive symptoms and suicidality at age 18 and 24.

	A	C	E	rA	rC	rE	rPh	%A	%C	%E
AGE 18										

OCS total	.37 (0.32, 0.41)	–	.63 (0.59, 0.68)						–	
Suicide attempts	.61 (0.48, 0.72)		.39 (0.28, 0.52)	.42 (0.29, 0.55)	–	.13 (0.02, 0.25)	.26 (0.22, 0.30)	74.6 (53.2, 96.3)		25.4 (3.7, 46.8)

Forbidden thoughts	.37 (0.29, 0.44)	–	.63 (0.56, 0.71)	.45 (0.29, 0.61)	–	.24 (0.09, 0.39)	.33 (0.28, 0.39)	64.0 (41.1, 86.7)	–	36.0 (13.3, 58.9)
Suicide attempts	.62 (0.49, 0.72)		.38 (0.28, 0.51)							

Symmetry	.35 (0.24, 0.42)	.01 (0.00, 0.09)	.64 (0.58, 0.70)							
Suicide attempts	.51 (0.16, 0.72)	.08 (0.00, 0.35)	.40 (0.28, 0.54)	.40 (0.50, 0.63)	1.00 (−1.00, 1.00)	.10 (−0.04, 0.25)	.25 (0.20, 0.30)	67.5 (6.7, 100.0)	11.5 (0.0, 31.0)	21.0 (0.0, 51.0)

AGE 24										

OCS total	.38 (0.31, 0.44)	–	.62 (0.56, 0.69)	.37 (0.21, 0.53)	–	.29 (0.17, 0.40)	.32 (0.28, 0.37)	49.6 (27.0, 70.7)	–	50.4 (29.3, 73.0)
Suicidality	.49 (0.36, 0.61)		.51 (0.39, 0.64)							

Obsessing	.23 (0.03, 0.42)	.11 (0.00, 0.26)	.65 (0.58, 0.73)							
Suicidality	.47 (0.28, 0.60)	.01 (0.00, 0.13)	.52 (0.40, 0.65)	.65 (0.35, 1.00)	1.00 (−1.00, 1.00)	.33 (0.21, 0.44)	.44 (0.41, 0.48)	48.2 (12.3, 76.3)	8.9 (0.0, 34.0)	42.9 (27.2, 60.1)

Ordering	.29 (0.21, 0.36)	–	.71 (0.64, 0.78)							
Suicidality	.49 (0.36, 0.61)		.51 (0.39, 0.64)	.32 (0.12, 0.51)	–	.15 (0.03, 0.26)	.21 (0.16, 0.25)	57.8 (22.9, 91.6)	–	42.2 (8.1, 77.1)

*Note:* OCS = obsessive-compulsive symptoms; *A* = additive genetic effects; *E* = non-shared environmental effect; rA = genetic correlation; rE = non-shared environmental correlation; rPh = phenotypic correlation;% *A* = percentage of phenotypic correlation accounted for by additive genetic factors;% *E* = percentage of phenotypic correlation accounted for by non-shared environmental factors. 95% confidence intervals in parentheses. Variability in the C estimates for suicide attempts at age 18 across the bivariate models results from different information being in each analysis from the cross-twin, cross-trait correlations.

**Fig. 1:**
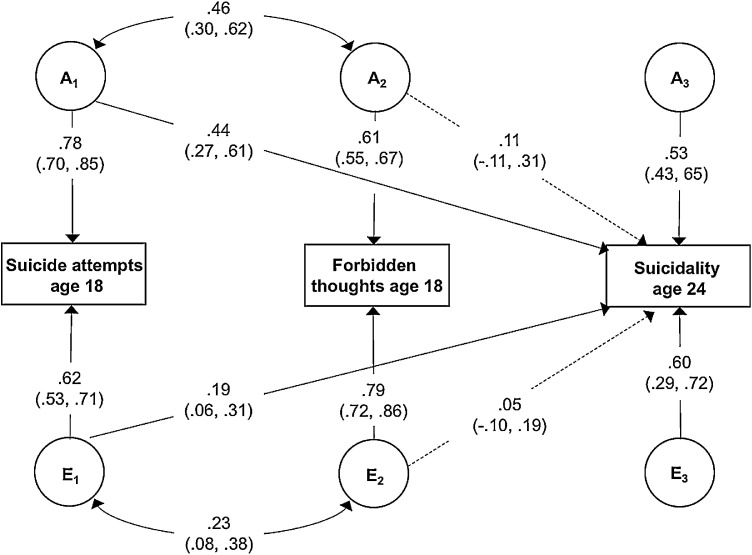
Genetic and environmental influences on the association suicidality and forbidden thoughts at age 18 with suicidality at age 24. Note: *A* = additive genetic effects; *E* = non-shared environmental effects; values are standardized, unsquared path estimates; 95% confidence intervals in parentheses. Variance components can be calculated by squaring the path estimate. This can be converted to a percentage by multiplying by 100. For example, the influence of A2 on forbidden thoughts at age 18 is 0.61^2^ x 100 = 37%.

A trivariate model was used to examine the aetiology of the prospective association between OCS at age 18 and suicidality at age 24, controlling for previous suicide attempts at age 18. This analysis was conducted using forbidden thoughts at age 18, since this was the only OCS variable at age 18 that had a correlation >0.2 with suicidality at age 24 (see Table S5). Estimates from the model are shown in Fig. 1. Genetic effects on suicidality at 18 had a substantial influence on suicidality at age 24. After accounting for genetic and environmental effects on suicidality at age 18, the additional genetic and non-shared influences on forbidden thoughts at age 18 had a small impact on suicidality at age 24. Genetic factors accounted for 63.7% (95% CIs 0.0 – 1.00) of the unique phenotypic association between OCS at age 18 and suicidality at age 24, with the remaining 36.3% (95% CIs 0.0 – 100.0) accounted for by non-shared environmental factors.

## 4 Discussion

To our knowledge, this is the largest investigation of OCS and suicidality in young adults to date, and the first to examine genetic and environmental influences on their association. Four key findings emerged. First, we found that total OCS were positively associated with concurrent reports of suicidal attempts at age 18 and suicidality (ideation and attempts) at age 24. This is consistent with findings from previous studies, which have primarily focused on adult OCD patients (Angelakis et al., 2015; Pellegrini et al., in press). Our findings extend previous research by demonstrating that the link between OCS and suicidality is evident in late adolescence and early adulthood, a period associated with the highest risk of suicidal thoughts and attempts (Piscopo et al., 2016). Furthermore, by examining continuously distributed OCS in a community sample, we demonstrate that the association between OCS and suicidality is not only evident in individuals with diagnosed OCD, as shown in previous research (Angelakis et al., 2015), but also applies across the OCS continuum. Importantly, we found that the association of total OCS with suicidality was attenuated but remained significant when adjusting for coexisting depression and anxiety symptoms. OCS may therefore be a unique risk factor for suicidality.

Second, we found that specific OCS dimensions had differential associations with suicidality. Forbidden thoughts and obsessing were positively and uniquely related to suicidality at ages 18 and 24, respectively. These subscales capture similar symptoms, including sexual, religious, and aggressive obsessions (Bejerot et al., 2014; Foa et al., 2002; Huppert et al., 2007). Our findings therefore indicate that such “taboo obsessions” are robustly linked with suicidality across developmental stages. This is consistent with previous studies showing that taboo obsessions are associated with suicidality amongst OCD patients (Balci and Sevincok, 2010; Khosravani et al., 2017; Kim et al., 2016; Storch et al., 2015; Torres et al., 2011; Velloso et al., 2016). We also found that symptoms relating to symmetry and ordering (e.g., arranging things in a special order) were positively associated with suicidality at age 18 but not age 24. This discrepancy could reflect the use of different measures across ages or, alternatively, genuine developmental differences may exist. Interestingly, in the current study we also found that higher levels of checking symptoms (e.g., excessive checking of doors, taps, light switches) were linked with *lower* risk of suicidality. This novel finding requires replication, but raises the possibility that some common OCS dimensions may be associated with reduced risk of suicidality, potentially leading to the false perception of OCD as a low risk disorder. Further research is needed to understand the mechanisms underpinning the differential links of OCS subtypes with suicidality. Such effects could be explained by differences in the aetiologies, neuropsychological profiles, associated temperaments, and burden of OCS subtypes. For example, it has been suggested that sexual and/or religious obsessions are more distressing than other forms of OCS, thereby increasing vulnerability to suicidality (Storch et al., 2015).

Third, we found that OCS at age 18 prospectively predicted suicidality over a six-year period, even when controlling for baseline suicide attempts. This novel finding is consistent with the notion that OCS is a risk factor for suicidality in youth. After adjusting for anxiety and depressive symptoms, we found that only forbidden thoughts remained a significant positive predictor of later suicidality. This is in keeping with the results of our cross-sectional analyses, showing that taboo obsessions are more robustly associated with suicidality than other OCS subtypes. Interestingly, when adjusting for baseline suicide attempts, anxiety, and depression in the prospective analyses, contamination symptoms at age 18 emerged as a significant *negative* predictor of suicidality. This again suggests that certain OCS may be linked with reduced risk of suicidality.

Fourth, our results indicate that much of the cross-sectional and longitudinal covariance between OCS and suicidality is driven by shared genetic vulnerability. For example, genetic effects accounted for 64% of the association between forbidden thoughts and suicidality at age 18, and 48% of the association between obsessing and suicidality at age 24. Importantly, across all genetic models, we found substantial non-shared environmental effects, explaining 21% to 50% of the association between OCS and suicidality. Of note, our results are comparable to findings from a previous family study, which estimated additive genetic and non-shared environmental influences on the familial coaggregation of OCD with suicide attempts at 60% and 40% respectively (Sidorchuk et al., 2019).

The current findings have several clinical implications. First, they highlight the importance of ongoing risk assessment and management in youth experiencing OCS. Coexisting anxiety and depression symptoms may contribute to, but are not the only indicator of, suicidality risk amongst individuals with OCS. Suicidality appears to be robustly linked to taboo obsessions, which are common amongst adolescents and adults with OCD (Fernández de la Cruz et al., 2013; Geller et al., 2001; Ruscio et al., 2010), but associated with high levels of shame and often concealed (Weingarden and Renshaw, 2015). Thus, it is crucial that clinicians directly ask about these symptoms. Second, our results suggest that the association between OCS and suicidality is largely due to a shared genetic vulnerability. The number and scale of genome-wide studies of OCD and suicidality have rapidly increased in recent years (Mattheisen et al., 2015; Sokolowski et al., 2014; Stewart et al., 2013). Our findings indicate that identifying the genetic architecture of OCS could elucidate biological mechanisms involved in suicidality, and vice versa. Third, our findings indicate that non-shared environmental factors also explain a significant proportion of the cross-sectional and prospective link between OCS symptoms and suicidality. This supports the view that suicidality can partially arise as a functional consequence of the burden of OCS, raising the possibility that effective treatment of OCS, particularly taboo obsessions, could ameliorate suicidality risk. Importantly, taboo obsessions are highly responsive to evidence-based OCD treatments (Fernández de la Cruz et al., 2013; Mataix-Cols et al., 1999; Storch et al., 2008).

## 5 Limitations

Strengths of this study include the large sample size and longitudinal design. However, the results should be interpreted in the context of some limitations. First, although participants who met diagnostic criteria for OCD were not excluded from this study, the focus was on OCS as a continuous trait, and our findings are not necessarily applicable to diagnosable OCD. However, OCD is recognised as being quantitatively but not qualitatively distinct from sub-clinical OCS (Abramowitz et al. 2014). Second, analyses were primarily based on self-report measures and therefore our estimates may have been influenced by common method variance. Of note, we found evidence of a significant association of self-reported OCS with parent-report suicidal ideation and attempts at age 18, although this became non-significant when adjusting for depressive symptoms. Third, OCS and suicidality were assessed with different measures at age 18 and 24, meaning that findings are not directly comparable across age. Furthermore, suicidal ideation and suicide attempts were assessed using single items taken from various measures. Further studies are needed incorporating more robust assessment of both suicidal thoughts and behaviours. Fourth, our longitudinal genetic model was not sufficiently powered, resulting in wide confidence intervals for parameter estimates. Estimates from this model should be taken as indicative rather than absolute. Fifth, there are limitations inherent to twin designs, as previously described (Plomin et al., 2013; Rijsdijk and Sham, 2002).

## 6 Conclusions

The current study demonstrates that certain OCS, particularly taboo obsessions, are strongly associated with suicidality during late adolescence and young adulthood. Moreover, these symptoms prospectively predict suicidality over time, even when controlling for suicide attempts at baseline. Importantly, both genetic and non-shared environmental influences appear to underpin the cross-sectional and longitudinal associations between OCS and suicidality. Taken together, our results raise the possibility that effectively detecting and treating OCS, especially taboo obsessions, could reduce risk for suicidality later in life.

## Declaration of Competing Interest

David Mataix-Cols receives royalties for contributing articles to UpToDate, Wolters Kluwer Health and for editorial work from Elsevier. Henrik Larsson has served as a speaker for Evolan Pharma, Eli-Lilly, and Shire, and has received research grants from Shire; all outside the submitted work. Lorena Fernández de la Cruz receives royalties for contributing articles to UpToDate, Wolters Kluwer Health. All other authors report no potential conflicts of interest.

## Appendix Supplementary materials

Image, application 1
