# Supplementary material for: Concurrent and prospective associations of obsessive-compulsive symptoms with suicidality in young adults: A genetically-informative study
Source: J Affect Disord. 2021 Feb 15;281:422–30. doi: 10.1016/j.jad.2020.10.065 (PMC7843953; doi:10.1016/j.jad.2020.10.065)
Supplement: Supplementary file 1 [file mmc1.docx]

**Table S1: Results of logistic regression testing the associations of age 18 variables with completion of OCS and suicidality measures at both ages 18 and 24.**

| **Age 18 variables** | **Odds ratio** | **z** |
| --- | --- | --- |
| Sex | 1.42 (1.25 – 1.62) | 5.35 |
| BOCS | .99 (.91 – 1.08) | -0.22 |
| Suicidality | .81 (.63 – 1.03) | -1.70 |
| CES-D | 1.02 (1.01 – 1.03) | 3.07 |
| SCARED | .80 (1.01 – 1.03) | -5.65 |
| *R*^2^ = .01 | | |

*Note:* BOCS = Brief Obsessive Compulsive Scale; CES-D = Center for Epidemiologic Studies Depression Scale; SCARED = Screen for Child Anxiety Related Emotional Disorders.

**Table S2: Results of Principal Component Analysis based on tetrachoric correlations of Brief Obsessive Compulsive Scale items.**

|  |  | **Component** | | | | |
| --- | --- | --- | --- | --- | --- | --- |
| **Item** | **Question** | **Symmetry** | **Forbidden thoughts** | **Contamination** | **Magical thoughts** | |
| 1 | I am worried about dirt, germs and virus. |  |  | .51 | |  |
| 2 | I wash my hands very often or in a special way to be sure I am not dirty or contaminated. |  |  | .50 | |  |
| 3 | I fear that my actions might harm others. |  | .43 |  | |  |
| 4 | I fear I will lose control and do something I don’t want to do. |  | .44 |  | |  |
| 5 | I have unpleasant forbidden or perverse sexual thoughts.  images or impulses that frighten me. |  | .45 |  | |  |
| 6 | I must check the stove or other electrical appliances,  that I have locked the door or make sure that things  have not disappeared. | .28 |  |  | |  |
| 7 | My dirty words, thoughts and curses directed towards  God bothers me; I have a fear of offending God. |  |  |  | | .68 |
| 8 | In order to prevent something terrible to happen I must have special thoughts or acts done in a special way. |  |  |  | | .46 |
| 9 | I am occupied with morality issues, justice or what is right or wrong. | .28 |  |  | |  |
| 10 | How things are placed or how they are positioned is important to me. It needs to feel “just right” (but isn’t associated with magical thinking). | .35 |  |  | |  |
| 11 | I get a compelling urge to put my things in a special order. | .32 |  |  | |  |
| 12 | I have a compelling urge to repeat certain actions until  it feels just right. | .32 |  |  | |  |

*Note:* Only highest factor loadings are shown. *N* = 9,175

**Table S3: Suicidality items administered at ages 18 and 24.**

|  | **Questionnaire source** | **Item** |
| --- | --- | --- |
| **CATSS-18** | |  |
|  | Adult Behavior Checklist  (parent-report) | [My child . .] Deliberately harms self or attempts suicide |
|  |  | [My child . .] Talks about killing self |
|  | Life History of Aggression  (self-report) | Have you deliberately attempted to kill yourself when you were angry or despondent? |
| **CATSS-24** | | |
|  | Center for Epidemiologic Depression Scale-Revised  (self-report) | [How often have you felt this way in the last two weeks . .] I wished I were dead |
|  | Suicidal Thoughts Questionnaire  (self-report) | Have you ever had thoughts about taking your life? |
|  |  | Have you ever made an attempt to take your life? |
|  |  | If "yes", did the attempt lead to that you went to a doctor, emergency room, or another health care facility? |
|  |  | If "yes", did the attempt lead to that you were hospitalized overnight or longer? |

**Table S4: Skewness of variables before and after log transformation.**

| **Scale** | **Raw variable** | **Log transformed variable** |
| --- | --- | --- |
| BOCS | 1.79 | .62 |
| OCI-R Total | 1.38 | -.48 |
| OCI-R washing subscale | 2.03 | .76 |
| OCI-R obsessing subscale | 1.80 | .65 |
| OCI-R ordering subscale | 1.17 | -.15 |
| OCI-R checking subscale | 1.50 | .11 |
| CESD | 1.82 | -.23 |
| SCARED | 1.15 | -.94 |

*Note:* BOCS = Brief Obsessive Compulsive Scale; OCI-R = Obsessive-Compulsive Inventory-Revised version; CESD = Center for Epidemiologic Depression Scale; SCARED = Screen for Child Anxiety Related Emotional Disorder.

**Table S5: Polychoric / biserial correlations of OCS and suicidality variables**

|  | Suicide attempts at age 18 | Suicidality at age 24 |
| --- | --- | --- |
| AGE 18 |  |  |
| BOCS total | **.26 (.22, .30)** | .19 (.13, .25) |
| Symmetry | **.25 (.20, .30)** | **.30 (.23, .37)** |
| Forbidden thoughts | **.33 (.09, .39)** | .11 (.02, .20) |
| Contamination | .15 (.10, .21) | .15 (.08, .22) |
| Magical thoughts | .18 (.11, .24) | .05 (-.03, .12) |
|  |  |  |
| AGE 24 |  |  |
| OCD-R total | – | **.32 (.28, .37)** |
| Ordering | – | **.21 (.16, .25)** |
| Obsessing | – | **.44 (.41, .48)** |
| Washing | – | .15 (.11, .20) |
| Checking | – | .11 (.11, .20) |

*Note:* The correlations between BOCS subscales and suicidality variables are polychoric. All other correlations are biserial. Associations shown in bold were decomposed using twin model fitting. BOCS = Brief Obsessive Compulsive Scale; OCI-R = Obsessive-Compulsive Inventory-Revised version. 95% confidence intervals in parentheses.

**Table S6: Fit comparisons for bivariate models at age 18.**

|  | **Base model** | **Comparison model** | **ep** | **-2LL** | **df** | **AIC** | **diffLL** | **diffdf** | ***p*** |
| --- | --- | --- | --- | --- | --- | --- | --- | --- | --- |
| Total OCS / suicide attempts |  |  |  |  |  |  |  |  |  |
|  | Saturated | N/A | 15 | 29159.35 | 17377 | -5594.65 | NA | NA | NA |
|  | Saturated | ACE | 13 | 29168.59 | 17381 | -5593.41 | 9.24 | 4 | .06 |
|  | Saturated | **AE** | **10** | **29168.66** | **17384** | **-5599.34** | **9.31** | **7** | **.23** |
|  | ACE | AE | 10 | 29168.66 | 17384 | -5599.34 | 0.07 | 3 | 1.00 |
| Forbidden thoughts / suicide attempts |  |  |  |  |  |  |  |  |  |
|  | Saturated | N/A | 17 | 15629.64 | 17574 | -19518.36 | NA | NA | NA |
|  | Saturated | ACE | 16 | 15643.14 | 17577 | -19510.86 | 13.50 | 3 | .004 |
|  | Saturated | **AE** | **13** | **15643.15** | **17580** | **-19516.85** | **13.50** | **6** | **.04** |
|  | ACE | AE | 13 | 15643.15 | 17580 | -19516.85 | .004 | 3 | 1.00 |
| Symmetry /  suicide attempts |  |  |  |  |  |  |  |  |  |
|  | Saturated | N/A | 17 | 20913.83 | 17501 | 14088.17 | NA | NA | NA |
|  | Saturated | **ACE** | **16** | **20918.40** | **17504** | **14089.60** | **4.57** | **3** | **.21** |

*Note:* ep= estimated parameters; -2LL = minus twice the log likelihood; df = degrees of freedom; AIC = Akaike’s information criterion; diffLL = difference in -2LL; diffdf = difference in df; A = additive genetic influence; E = non-shared environmental influences. Bold typeface indicates final model selected.

**Table S7: Fit comparisons for bivariate models at age 24.**

|  | **Base model** | **Comparison model** | **ep** | **-2LL** | **df** | **AIC** | **diffLL** | **diffdf** | ***p*** |
| --- | --- | --- | --- | --- | --- | --- | --- | --- | --- |
| Total OCS / suicidality |  |  |  |  |  |  |  |  |  |
|  | Saturated | N/A | 26 | 13523.65 | 6311 | 901.65 | NA | NA | NA |
|  | Saturated | ACE | 13 | 13552.98 | 6326 | 900.98 | 29.33 | 15 | .01 |
|  | Saturated | **AE** | **10** | **13552.98** | **6329** | **894.98** | **29.33** | **18** | **.04** |
|  | ACE | AE | 10 | 13552.98 | 6329 | 894.98 | -3.42e-09 | 3 | 1.00 |
| Obsessing / suicidality |  |  |  |  |  |  |  |  |  |
|  | Saturated | NA | 26 | 12609.15 | 6348 | -86.85 | NA | NA | NA |
|  | Saturated | **ACE** | **13** | **12638.16** | **6363** | **-87.84** | **29.00** | **15** | **.02** |
|  |  |  |  |  |  |  |  |  |  |
| Ordering / suicidality |  |  |  |  |  |  |  |  |  |
|  | Saturated | NA | 26 | 12741.51 | 6356 | 29.51 | NA | NA | NA |
|  | Saturated | ACE | 13 | 12768.77 | 6371 | 26.77 | 27.27 | 15 | .03 |
|  | Saturated | **AE** | **10** | **12768.77** | **6374** | **20.77** | **27.27** | **18** | **.07** |
|  | ACE | AE | 10 | 12768.77 | 6374 | 20.77 | -1.58e-09 | 3 | 1.00 |

*Note:* ep= estimated parameters; -2LL = minus twice the log likelihood; df = degrees of freedom; AIC = Akaike’s information criterion; diffLL = difference in -2LL; diffdf = difference in df; A = additive genetic influence; E = non-shared environmental influences. Bold typeface indicates final model selected.

**Table S8: Fit comparisons for trivariate model of suicidality and forbidden thoughts at age 18, and suicidality at 24.**

| **Base model** | **Comparison model** | **ep** | **-2LL** | **df** | **AIC** | **diffLL** | **diffdf** | ***p*** |
| --- | --- | --- | --- | --- | --- | --- | --- | --- |
|  |  |  |  |  |  |  |  |  |
| Saturated | N/A | 29 | 18249.26 | 21275 | -24300.74 | NA | NA | NA |
| Saturated | ACE | 28 | 18267.45 | 21279 | -24290.55 | 18.19 | 4 | .001 |
| Saturated | **AE** | **22** | **18267.44** | **21285** | **-24302.56** | **18.18** | **10** | **.052** |
| ACE | AE | 22 | 18267.44 | 21285 | **-24302.56** | -.01 | 6 | 1 |

*Note:* ep= estimated parameters; -2LL = minus twice the log likelihood; df = degrees of freedom; AIC = Akaike’s information criterion; diffLL = difference in -2LL; diffdf = difference in df; A = additive genetic influence; E = non-shared environmental influences. Bold typeface indicates final model selected.
